# Fasting the mitochondria to prevent neurodegeneration: the role of ceramides

**DOI:** 10.3389/fnins.2025.1602149

**Published:** 2025-06-04

**Authors:** Luis Armando Valenzuela-Ahumada, Octavio Fabián Mercado-Gómez, Rubi Viveros-Contreras, Rosalinda Guevara-Guzmán, Alberto Camacho-Morales

**Affiliations:** ^1^Department of Biochemistry, College of Medicine, Universidad Autónoma de Nuevo León, Monterrey, Mexico; ^2^Department of Physiology, College of Medicine, Universidad Nacional Autónoma de México, Mexico City, Mexico; ^3^Centro de Investigaciones Biomédicas, Universidad Veracruzana, Xalapa, Mexico

**Keywords:** neurodegeneration, microglia, intermittent fasting, ceramides, mitophagy

## Abstract

Neurodegenerative diseases affect up to 349.2 million individuals worldwide. Preclinical and clinical advances have documented that altered energy homeostasis and mitochondria dysfunction is a hallmark of neurological disorders. Diet-derived ceramides species might target and disrupt mitochondria function leading to defective energy balance and neurodegeneration. Ceramides as bioactive lipid species affect mitochondria function by several mechanism including changes in membrane chemical composition, inhibition of the respiratory chain, ROS overproduction and oxidative stress, and also by activating mitophagy. Promising avenues of intervention has documented that intermittent fasting (IF) is able to benefit and set proper energy metabolism. IF is an eating protocol that involves alternating periods of fasting with periods of eating which modulate ceramide metabolism and mitochondria function in neurons. This review will address the detrimental effect of ceramides on mitochondria membrane composition, respiratory chain, ROS dynamics and mitophagy in brain contributing to neurodegeneration. We will focus on effect of IF on ceramide metabolism as a potential avenue to improve mitochondria function and prevention of neurodegeneration.

## Graphical abstract

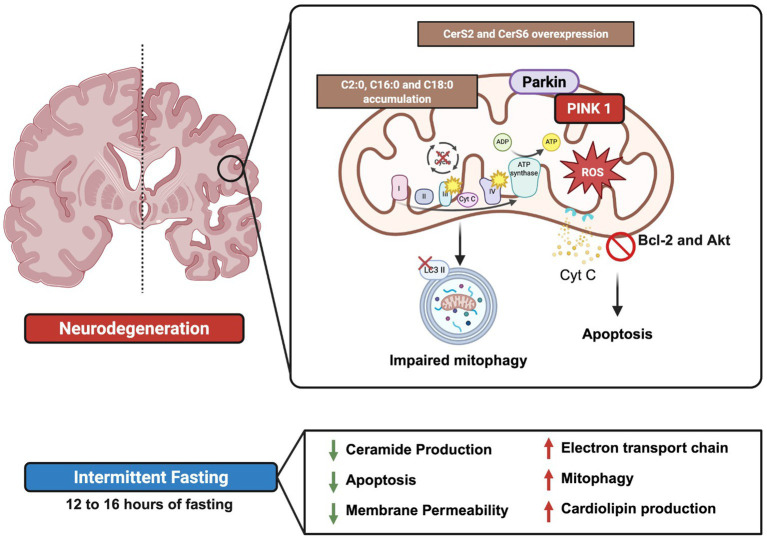

## 1 Introduction

Brain is a major body organ that integrate several neurodegenerative diseases including Alzheimer disease (AD), Parkinson disease (PD), primary tauopathies, frontotemporal dementia (FTD), amyotrophic lateral sclerosis (ALS), synucleinopathies (such as Lewy body dementia [LBD] and multisystem atrophy [MSA]), polyglutamine-related diseases (Huntington disease (HD) and spinocerebellar ataxias [SCA]), prion disease (PrD), traumatic brain injury (TBI), chronic traumatic encephalopathy (CTE), spinal cord injury (SCI), and multiple sclerosis (MS) (Wilson et al., 2023). According to the incidence, prevalence, and social impact up to 349.2 million individuals were affected by major neurological disorders worldwide (Ding et al., 2022; Huang et al., 2023). Recent reports documented the eight hallmarks of neurological disorders such as: pathological protein aggregation, synaptic and neuronal network dysfunction, aberrant proteostasis, cytoskeletal abnormalities, DNA and RNA defects, inflammation, neuronal cell death and altered energy homeostasis and mitochondria function (Wilson et al., 2023). While multifactorial triggers contribute to susceptibility to neurological disorders, defective in brain energetic deeply affect brain function. Accordingly, brain demands a high energy supply provided by glucose metabolism and mitochondria supporting synaptic transmission and maintain the plasma resting potential (Camacho and Massieu, 2006; Hamzeh et al., 2023). A decrease in glucose supply or pharmacologic inhibition of glucose or mitochondria metabolism into the brain, result in neurodegeneration (Shichkova et al., 2024; Camacho et al., 2006, 2007; Kazemeini et al., 2024).

Preclinical and clinical models documented that diet-derived ceramides might target and disrupt mitochondria function leading to neurodegeneration (Hamzeh et al., 2023). Ceramides are bioactive lipid species that serve as precursors of all complex sphingolipids (SLs) (Bernal-Vega et al., 2023). Ceramides are structural components in cell and mitochondrial membranes and modulates several cellular signaling events such as cell growth, cycle, death, and senescence, inflammation, immune modulation, cell adhesion, and migration, autophagy and stress response (Bernal-Vega et al., 2023). We and others have reported that ceramides affect mitochondria function favoring changes in membrane chemical composition, inhibition of the respiratory chain, overproduction of ROS reducing intracellular ATP production and causing oxidative stress and mitophagy in brain (Camacho-Morales et al., 2024). Accordingly, subjects diagnosed with neurodegenerative diseases have reported ceramide accumulation. For instance, plasma ratio of very long (C22-24) to long (C16-18) ceramides were found in AD susceptibility (McGrath et al., 2020). Also, elevated Cer species were found in the lesion area at early stages of AD (Han et al., 2002; Chowdhury et al., 2022; Reveglia et al., 2023). This evidence supports the notion that ceramides lipid species promote mitochondria dysfunction in brain which potentially might contribute to neurodegenerative susceptibility in subjects.

Intermittent fasting (IF) is an eating protocol that involves alternating periods of fasting with periods of eating and has recently gained popularity as a strategy for reducing body weight (Erdem et al., 2022; Steger et al., 2021). Furthermore, IF has been reported to decrease circulating levels of inflammatory markers in healthy subject (Faris et al., 2012; Perez-Kast and Camacho-Morales, 2025), as well as the inflammatory state associated with obesity (Zouhal et al., 2020). Also, IF decrease plasma levels of ceramide C17, C22, and C24 sphingomyelin, as well as C20, C22, C24, and C24:1 dihydrosphingomyelin (Madkour et al., 2023). IF also regulate the peroxisome proliferator-activated receptor *γ* coactivator 1α (PGC-1α), a transcription factor of mitochondria function, in neurons (Wilhelmi de Toledo et al., 2020; Mattson et al., 2018). IF upregulate the brain-derived neurotrophic factor (BDNF), favoring neurogenesis, synaptic plasticity and mitochondrial biogenesis (Mattson et al., 2018). This evidence supports that IF can prevent inflammatory response and enhance mitochondria functionality in individuals with chronic inflammation of elevated risk of poor cognitive function.

In this review, we will address the effect of IF on ceramide metabolism linked to mitochondria dynamics in brain and its potential effects on neurodegeneration. We will focus on the effect of ceramide assisting lipid composition of the mitochondria membrane, mitochondrial respiratory chain activity, ROS overproduction, oxidative stress and changes in mitophagy – apoptosis, and how these are mutually related. We aim this review within the framework of neurodegenerative diseases, which integrate the highest incidence and prevalence of mental illnesses in the population. The manuscript will describe findings in animal models (mainly rats and mice) and contrasting them with what has been reported so far in humans.

## 2 Ceramide in brain function and neurodegeneration

Ceramides are a family of bioactive lipids composed of a long-chain sphingoid base linked to a fatty acid chain through an amide bond. They vary in structure based on the length, hydroxylation, and saturation levels of both the sphingoid base and the fatty acid residues (Alonso and Goñi, 2018). Ceramides are predominantly found in the liver, adipose tissue, and *neural tissue*, where they modulate several signaling pathways. Three major metabolic pathways are involved in ceramide biosynthesis: the *de novo,* the salvage pathway and the sphingomyelinase pathway. The *De Novo* Pathway is located on the cytoplasmic surface of the endoplasmic reticulum (ER) and integrate the most important source of ceramides. The *De Novo* Pathway begins with the condensation of L-serine and a fatty-acyl CoA such as palmitoyl-CoA, myristoyl CoA, or stearoyl CoA at the endoplasmic reticulum catalyzed by the enzyme serine palmitoyltransferase (SPT) to form 3-ketosphinganine, which is then reduced to dihydrosphingosine by the NADH-dependent 3-ketosphinganine reductase (KDSR). Ceramide synthases (CerS) catalyze the acylation of dihydrosphingosine to dihydroceramide. According to their carbon chain lengths, six CerS isoforms have been reported in mammals displaying selective specificity for acyl CoAs. CerS1 attaches the C18 fatty acyl CoA (long chain); CerS2 exhibits activity toward the very long acyl CoAs such as C22–C24; CerS3 attaches the ultra-long fatty acyl CoA as C26, CerS4 specifies the C18–C20 CoA; and CerS5 and 6 have specificity for the C14–C16 CoA (Bernal-Vega et al., 2023). Ceramide synthesis is responsive to tissue-dependent distribution of ceramide synthases. For instance, CerS1 is mainly expressed in the brain, CerS2 is highly abundant in the liver and kidney, high ex- pression of CerS3 is found in the testis and skin, and elevated levels of CerS4 mRNA are found in the skin, heart, liver, and leukocytes, CerS5 is mainly expressed in lung epithelia and brain, whereas CerS6 show high expression in the intestine (Brachtendorf et al., 2019; Levy and Futerman, 2010; Bernal-Vega et al., 2023). Interestingly, Canals and Clarke (2022) found that mRNA expression levels of CerS do not directly correlate with the concentrations of ceramides with specific chain lengths. Finally, ceramide is produced by a desaturation reaction assisted by dihydroceramide desaturase (DDase) (Quinville et al., 2021; Bernal-Vega et al., 2023). This evidence suggests that ceramides synthesis is responsive to tissue-dependent distribution of enzymes and which are post-transcriptionally modulated by unknown mechanisms (Mullen et al., 2012).

In the salvage pathway, ceramide is generated from partial degradation of membrane glycolipids by glucosylceramidase (GCase). Also, in the sphingomyelin pathway complex sphingolipids are hydrolyzed by sphingomyelinases (SMase) in lysosomes. Next, ceramide is transported to the Golgi complex through vesicular and non-vesicular protein ATP-dependent coupling allowing the production of complex SLs. Finally, ceramide can follow four major catabolic pathways: (1) phosphorylated by the ceramide kinase (CERK) to form ceramide-1-phosphate (C1P), (2) incorporated into sphingomyelin-by-sphingomyelin synthase (SMS) activity, (3) or into glucosylceramide by the activation of glucosylceramide synthases (GCS), leading to the generation of complex SLs like cerebrosides, gangliosides, sulfatides and globosides; and (4) ceramides are deacylated by the ceramidases (CDase) to produce sphingosine. Sphingosine kinases (SKs) phosphorylate sphingosine to produce sphingosine-1-phosphate (S1P), which is degraded by sphingosine phosphate phosphatase (SPP) or S1P lyase synthesizing. The genes encoding CerS enzymes differ in their spatial and temporal expression patterns, as well as in their ability to produce ceramides with varying chain lengths. Although all identified CerS enzymes exhibit similar Km values (affinity for substrates), they vary in their selectivity for acyl-CoAs depending on the length of the acyl chain (Bernal-Vega et al., 2023).

**Figure 1:**
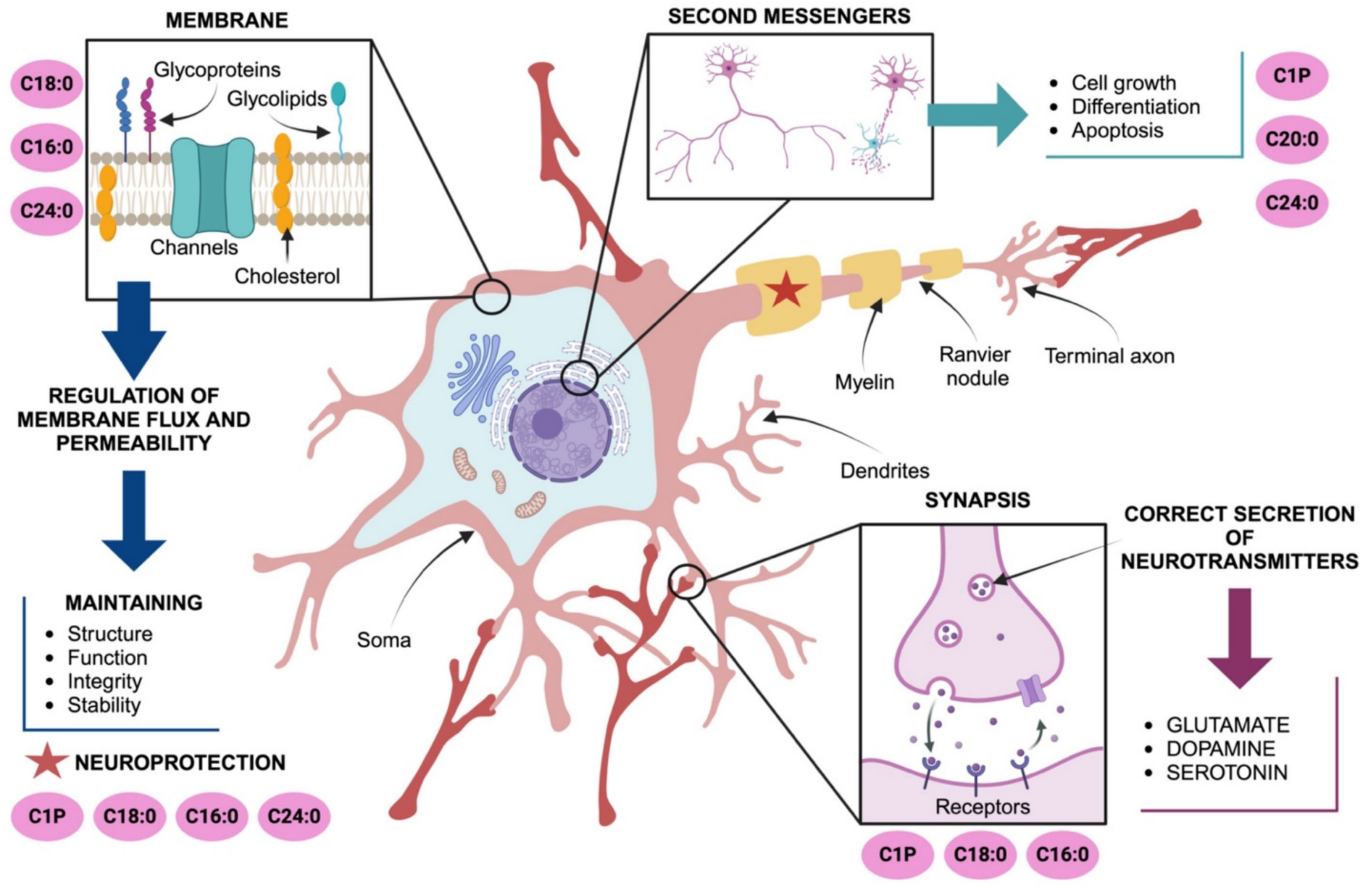
Ceramides modulate neuronal development, function, and protection. C18:0, C16:0 and C24:0 maintain plasma membrane integrity and fluidity. C20:0, C24:0 and ceramide-1-phosphate (C1P) are also involved in the fine regulation of processes including cell growth, differentiation, neuroplasticity and apoptosis. C24:0 ceramide supports myelination and axonal health, indirectly protecting neuronal endings and facilitating signal propagation to synaptic terminals. Ceramides, particularly C16:0, C18:0, and C24:0, play significant roles in modulating neurotransmitter release coordinating excitotoxicity and ensuring proper neuronal communication. Abbreviations: ceramide-1-phosphate (C1P).

The role of ceramides in the brain is essential for maintaining the structure and function of cell membranes, contributing to their integrity, fluidity and stability (Mencarelli and Martinez-Martinez, 2013). For instance, C24:0 ceramide maintains cell membrane integrity by enhancing lipid packing and promoting a more rigid membrane structure, thereby regulating permeability. Conversely, replacing C24:0 ceramide with shorter-chain ceramides like C16:0 increased membrane permeability, underscoring the importance of very long-chain ceramides in barrier function (DiPasquale et al., 2022; Ventura et al., 2020). In fact, ceramides are expressed in oligodendrocytes, where they support myelin integrity. The ceramides that serve as precursors for complex sphingolipids essential to myelin sheath formation. Initial reports documented active sphingolipid metabolism of C2:0 and C6:0 ceramides to Sph and S1P in differentiated oligodendrocytes (Qin et al., 2010). Exogenous S1P is efficiently metabolized back to Sph and predominantly C18-ceramide supporting myelin confirmation. Oligodendrocytes also accumulate sphingomyelin and GalCer and C22:0, C24:0 and C24:1 ceramides are found in oligodendrocytes (Hammerschmidt and Brüning, 2022) (Figure 1).

Beyond these structural roles, ceramides are also involved in intracellular signaling, acting as second messengers in critical processes such as cell growth, differentiation, apoptosis, and stress responses essential for neuronal survival and synaptic function. C16:0 ceramide maintains synaptic integrity and plasticity, especially under conditions of metabolic stress or excess fatty acids assuring memory and learning (Furuya et al., 1998). Also, C20:0 and C24:0 ceramides are critical during neuronal development maintaining neuronal networks, modulating neurite outgrowth and axon guidance (Olsen and Færgeman, 2017; Chaurasia et al., 2019; Mullen et al., 2012). The ceramide-1-phosphate (C1P), a phosphorylated derivative of ceramide, favors cell survival, proliferation, and anti-apoptotic signaling through the activation of PI3K/Akt, ERK1/2, and NF-κB pathways. C1P also modulates inflammatory responses and inhibits acid sphingomyelinase activity supporting neuronal survival (Furuya et al., 1998; Gómez-Muñoz, 2006). C1P and C2 ceramides also regulate the dynamics of the cytoskeleton and axonal guidance by their effects on G-protein coupled receptors coupled to Rho GTPases signaling. Additionally, C1P modulates the activity of neurotrophic factors, such as BDNF (brain-derived neurotrophic factor), which are vital for axon growth, survival, and synaptic plasticity (Furuya et al., 1998; Gómez-Muñoz, 2006) (Figure 1).

Furthermore, in neurons ceramides regulate neurotransmitter release, particularly modulating glutamate, dopamine, and serotonin, which are vital for neuronal communication and synaptic plasticity (Farsi et al., 2021; López et al., 2025). Under normal conditions, C16:0 ceramide, produced by Ceramide Synthase 6 (CerS6), plays a crucial role in synaptic glutamate release by organizing lipid rafts configuration assuring clustering of the soluble N-ethylmaleimide-sensitive factor attachment protein receptor (SNARE) complex and vesicle fusion (Sapoń et al., 2023). In fact, C16:0 acts as a second messenger, activating protein kinase C (PKCζ) and promoting Ca^2+^-dependent exocytosis of glutamate (Wheeler et al., 2009), supporting short-term plasticity. C18:0 ceramide is enriched in hippocampal and cortical neurons (Qin et al., 2010) where higher basal levels of C18:0 ceramide correlate with efficient dopamine release and signaling (Schoffelmeer et al., 2000). C1P regulates the function of serotonin receptors and influence serotonin-related cellular signaling (Ji et al., 2011). C1P may also influence serotonin production by regulating the activity of tryptophan hydroxylase possibly through changes in cellular stress responses or phosphorylation (Sinenko et al., 2023). These evidences documented the role of C16:0, C18:0, and C1P:0 on neurotransmitter release and synaptic plasticity.

Dysregulation of ceramide metabolism in the brain has been linked to several brain pathologies, including neurodegenerative diseases such as Alzheimer’s and Parkinson’s, and psychiatric disorders like anxiety disorders, depression, and schizophrenia, and neurological injuries (Sambolín-Escobales et al., 2022; Schumacher et al., 2022; Mielke et al., 2013; Liu et al., 2024; Afridi et al., 2022). Ceramides promote neuroprotection by modulating stress, including oxidative stress, inflammation, and excitotoxicity in neurons and glia. In neurons, ceramides support the coating of neuronal endings, safeguarding synaptic connections (Mencarelli and Martinez-Martinez, 2013). Ceramides are also expressed in glia cells. In a recent report McInnis et al. (2024) documented high expression of C24:0 ceramide in astrocytes and C24:1 in microglia when compare with C16:0 and C18:0 ceramides in neurons. Also, S1P modulates the translocation of NF-κB to the nucleus in astrocytes (Rajagopalan et al., 2015) leading to experimental autoimmune encephalomyelitis (Linnerbauer et al., 2020). LacCer activates the IRF-1 and NF-κB expression favoring proinflammatory gene profiles in astrocytes (Giovannoni and Quintana, 2020). Accordingly, exposure microglia to palmitic acid (C16:0/PA) enhanced the expression of proinflammatory markers (Duffy et al., 2015), and a decreased in C22:0–C24:0 ceramides by deletion of CerS2 triggered the activation of microglial cells and progressive myelin atrophy (Teo et al., 2023). Potential molecular mechanisms contributing to demyelination and neurodegeneration suggest that ceramides C18:0, C22:0, C24:0 and C24: activate the neuronal NADPH oxidase 2 (NOX2)1 (Arsenault et al., 2021) or endoplasmic reticulum stress response (García-González et al., 2018). These evidences support the role of ceramides on glia function.

Finally, ceramides might be synthetized by mitochondria itself. For instance, initial reports in murine models identified three ceramide synthase subtypes CerS1/2/6 in the mitochondria of mouse brain (Novgorodov et al., 2011; S. Ding et al., 2024). In fact, CerS2 co-localizes with the translocase of outer mitochondrial membrane 20 (TOM20) on the outer mitochondria membrane and the CerS6 associates with the adenine nucleotide translocase (ANT) on the inner mitochondria membrane (Yabu et al., 2009; Ding et al., 2024). In addition, mitochondria also recruit additional enzymes related to ceramide metabolism including neutral sphingomyelinase (nSMase) (Novgorodov et al., 2011; Ding et al., 2024) and neutral ceramidase (Rajagopalan et al., 2015; Ding et al., 2024). This suggests that mitochondria by itself integrate the enzymatic machinery to properly regulate ceramides and assist its biological activity potentially by its interaction to ER.

Accumulation of selective ceramides species might target and disrupt mitochondria function leading to neurodegeneration (Lima et al., 2023). In an elegant preclinical report authors documented accumulation of long-chain and very long-chain ceramides in brain of 5xFAD mice, which was associated with mitochondrial dysfunction (Crivelli et al., 2024). This evidence suggest that defective ceramide metabolism might target mitochondria favoring neurodegeneration. We next describe the role of ceramides on mitochondria dysfunction assisting neurodegeneration.

## 3 Ceramides and mitochondrial damage

Mitochondria are dynamic organelles that use oxidative phosphorylation to supply energy by producing adenosine triphosphate, which is indispensable for life and health in most eukaryotic organisms (Luo et al., 2020). Mitochondria display critical roles including metabolic homeostasis, redox signaling, regulation of Ca2+, mitochondrial-nuclear communication and cell death (Chen et al., 2023). When mitochondrial dysfunction occurs in cells, homeostasis is coordinating by mitophagy which balance a physiological response of mitochondrial biogenesis and clearance of damaged mitochondria (Luo et al., 2020). In fact, mitochondrial turnover and dynamics are precisely coordinated by a balance between fission—fusion processes (Chen et al., 2023).

Dysregulation of mitochondrial dynamics is one key pathogenic mechanism of diverse human pathologies including neurodegenerative diseases (Luo et al., 2020). Accordingly, ceramides species might target mitochondria in brain favoring susceptibility to neurodegeneration. Major advances addressing the role of mitochondria on neurodegenerative diseases have been mainly reported in two neurodegenerative diseases, Parkinson and Alzheimer (Alecu and Bennett, 2019). Although the precise molecular mechanisms remain largely elusive, we will describe how ceramides target mitochondria function in brain by several mechanisms: (1) changes in the lipid composition of the mitochondria membrane, (2) alteration in mitochondrial respiratory chain activity and increased in ROS production, and (3) changes in mitophagy—apoptosis process.

### 3.1 Ceramides affects the lipid composition of the mitochondria membranes

**Figure 2:**
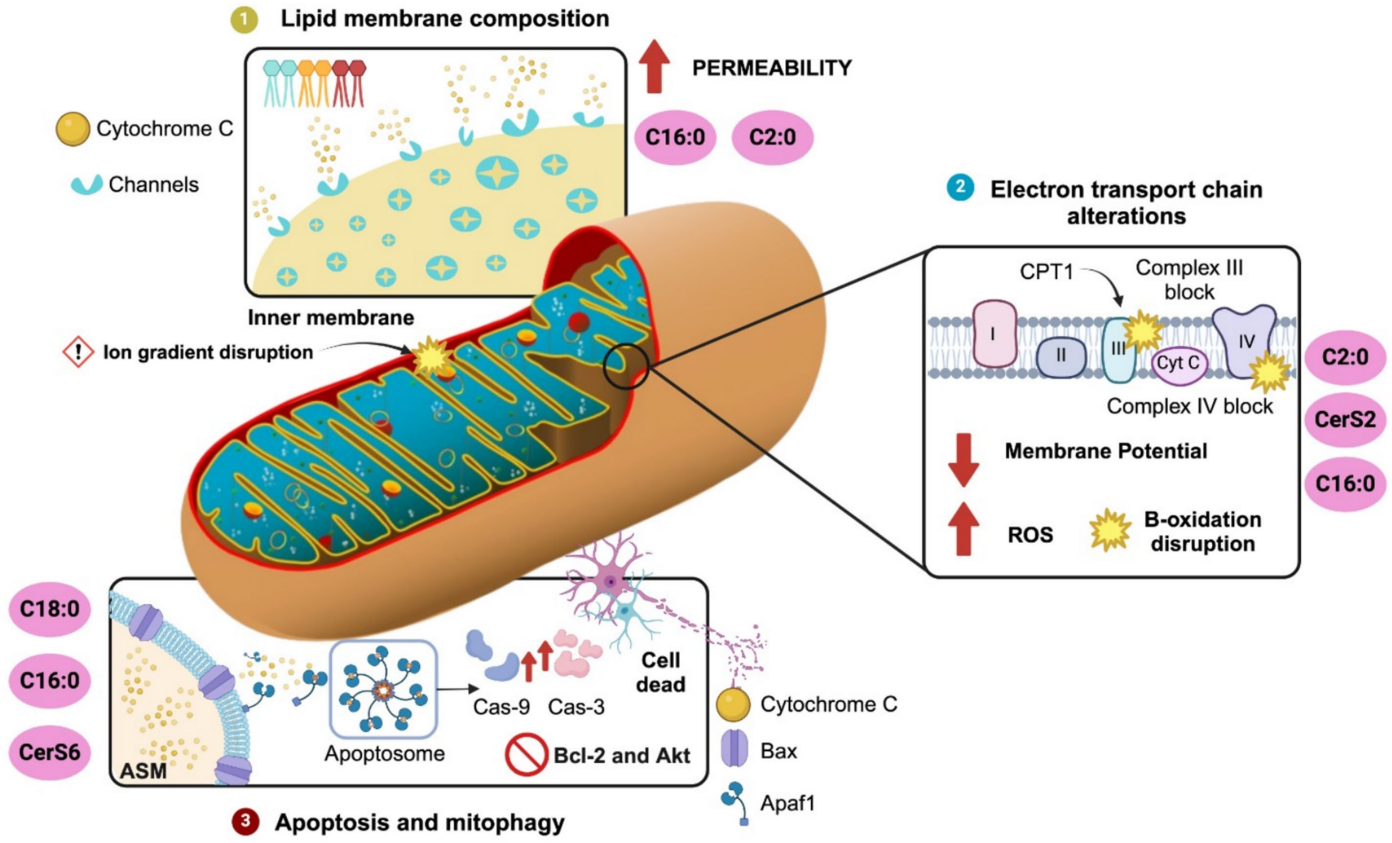
Ceramide-driven mitochondrial regulation. Ceramides modulates mitochondria dynamics by several pathways. (1) Lipid membrane composition: C2:0 and C16:0 ceramides increase mitochondrial membrane permeability by integrating channels into the inner mitochondria membrane assisting cytochrome c release. (2) Disruption of electron transport chain: C2:0 and C16:0 ceramides or CerS2 overexpression promoted ROS production via mPTP interaction and Complex III inhibition (antimycin A site). Also, C16:0 inhibits Complex IV and CPT1, disrupting β-oxidation. (3) Ceramides activates apoptosis and mitophagy: C16:0 and C18:0 promoted Bcl-2 depletion and Akt inhibition and activated apoptosis by MOMP through Bcl-xL-sensitive channel formation and interaction with Bax/VDAC. C18:0 ceramide, produced by CerS1 transported via p17/PERMIT, triggers mitophagy through LC3B-II independently of caspases. ASM and ceramides coordinate mitophagosome formation. Conversely, CerS6 impairs mitophagy via SQSTM1 interaction. Ceramide synthase 2, CerS2; Ceramide synthase 6, CerS6.

Mitochondrial membrane lipids are essential for several mitochondria processes such as membrane architecture, transport of proteins into the matrix, activity of respiratory proteins, mitochondrial fission and fusion, and contribution of lipids to endoplasmic reticulum related through contacts by the mitochondria-associated membranes (MAMs) (Böttinger et al., 2016; Aufschnaiter et al., 2017). The major lipid species in mitochondria included up to 40% phosphatidylcholine (PC), 27% phosphatidylethanolamine (PE), 3% phosphatidylserine (PS), 15% phosphatidylinositol (PI), 2% phosphatidic acid (PA) and 13% cardiolipin (CL) (Aufschnaiter et al., 2017). Lipidomic profiles into the outer mitochondrial membrane, included up to 46% PC, 33% PE, 1% PS, 10% PI, 2% PA and 6% CL; whereas in the inner mitochondrial membrane it is found 38% PC, 24% PE, 4% PS, 16% PI, 2% PA and 16% CL (Aufschnaiter et al., 2017). Notably, ceramides content in mitochondria is located up to three-fold higher in the outer rather than in the inner mitochondrial membrane, which potentially might regulate the formation of protein-permeable channels (Siskind and Colombini, 2000). MAMs also integrates a subcellular compartmentalization of ceramide metabolism between mitochondria and endoplasmic reticulum capable of modulate cellular responses (Bionda et al., 2004). In fact, in an elegant study authors proposed an evolutionary scenario to explain mitochondria lipid metabolism, suggesting that mitochondria are outcomes of a merger between (alphaproteo-) bacteria and archaea showing ancestral combination of two main genes for lipid synthesis: cardiolipin and ceramide (Geiger et al., 2023). This evidence confirms that lipidomic profile and ceramide content in mitochondria membranes might modulate cellular responses and contributes to cellular dysfunction by disrupting mitochondrial integrity, morphology and function (Figure 2).

Initial reports documented that changes in ceramide species might affect lipid composition in the mitochondrial membrane. For instance, C16:0 and C2:0-ceramides might increase the permeability of mitochondria membranes releasing cytochrome c tentatively by channel formation (Siskind and Colombini, 2000). Mitochondrial permeability disrupts the ion gradients across the inner mitochondrial membrane, resulting in mitochondrial depolarization, a loss of oxidative phosphorylation, and an increase in ROS production (García-Ruiz et al., 1997). Mitochondrial permeability might be also facilitated by external factors such as mitochondrial calcium loading, pH changes, thiol oxidation, and activation of the adenine nucleotide translocator (ANT). In fact, researcher identified that mitochondrial permeability transition pore (mPTP) was sensitive to C2 ceramide incubation by promoting ROS overproduction (García-Ruiz et al., 1997). The accumulation of C16:0 ceramide also might alter the balance between sphingolipids, phospholipids, and other membrane constituents, leading to increased membrane rigidity and permeability (Fucho et al., 2017). In isolated liver mitochondria, C16:0 ceramides formed much higher membrane channels than the C22:0 ceramide (Stiban and Perera, 2015). It is proposed that membrane channels are formed from columns of ceramides that arrange in an anti-parallel fashion making a cylindrical shape spanning the hydrophobic interior of the mitochondrial outer membrane. Each column is composed of six Cer molecules associated to the MOMP and mitochondria permeabilization (Abou-Ghali and Stiban, 2015). The formation of ceramide-induced pores has been associated with interactions between ceramides and proteins such as Bax or the voltage-dependent anion channel (VDAC), which facilitate cytochrome c release and initiate apoptosis (Siskind et al., 2006; Pastorino et al., 2002) (Figure 2).

Finally, changes in the lipidomic profile of mitochondria membranes might compromise mitochondria permeabilization and cellular responses susceptible to neurodegeneration. A recent report documented accumulation of the ceramide precursor sphingosine and C20:0, C22:0, C22:1, C24:0 and C24:1 ceramide species in mitochondria isolated from both 5xFAD mice and AD patient brains (Crivelli et al., 2024). Also, defective CL synthesis, a lipid specie of ceramide metabolism, might alter mitochondrial cristae structure reported in AD and PD’s diseases (Argueti-Ostrovsky et al., 2024). Conversely, pharmacologic inhibition of the ceramide synthesis improved mitochondria morphology in lymphoid cells from patients with HD (Ciarlo et al., 2012) (Figure 2).

Together, changes in ceramide metabolism appears to modulate mitochondria membrane structure in neurodegenerative diseases. It is expected that membrane structure associates to mitochondrial dynamics and metabolism, we will address this topic in the next sections.

### 3.2 Ceramides affect the electron transport chain (ETC) and promote ROS generation

Mitochondria integrates the ETC to the oxidative phosphorylation to regulate cellular energy metabolism. The protein machinery of the ETC is composed of five enzyme complexes and two electron mobility carriers including the complex I (CI, NADH–ubiquinone oxidoreductase), complex II (CII, succinate dehydrogenase, SxDH), complex III (CIII, cytochrome bc1 oxidoreductase), complex IV (CIV, cytochrome c oxidase), ATP synthase, and the electron mobility carriers ubiquinone and cytochrome c (Kobayashi et al., 2023). Functionally, reducing equivalents from NADH and FADH provide electrons to the ETC which are finally accepted by oxygen to generate ATP. Accordingly, ROS such as the superoxide anion production originates from the Complex I and III, and can damage proteins, lipids or nucleic acids within the mitochondria and cell (Hadrava Vanova et al., 2020). Overexpression of CerS2 has been reported to reduce mitochondrial membrane potential compared to control groups (Qiu et al., 2018). Experimental evidence indicates that ceramide accumulation can reduce mitochondrial membrane potential by 30–50% in specific cell types (Novgorodov et al., 2008). This decline can be detected using fluorescent dyes such as TMRM or JC-1, which show a decrease in fluorescence when mitochondrial potential drops. Notably, C2-ceramide has been shown to induce a rapid reduction in mitochondrial membrane potential. For example, in rat cardiomyocytes, C2-ceramide caused a marked decrease in mitochondrial membrane potential, accompanied by fragmentation of the mitochondrial network (Parra et al., 2013). Further mechanistic insights were provided by Novgorodov et al. (2016), who explored the interaction between SIRT3, a mitochondrial deacetylase, and ceramide synthases (CerS1, CerS2, CerS6) in cerebral mitochondria. Their investigation using SIRT3 knockout mice revealed that the absence of SIRT3 led to increased mitochondrial ROS production and protein carbonylation, markers of oxidative stress and protein damage (Figure 2).

Several reports have documented the effect of ceramides accumulation on ETC and mitochondrial function in peripheral tissues and brain. For example, accumulation of ceramides in the mitochondria of skeletal muscle cells showed loss of the mitochondrial respiratory chain components, resulting in mitochondrial dysfunction and insulin resistance (Diaz-Vegas et al., 2023). Also, C2:0 ceramide block the activity of Complex III favoring ROS production, tentatively by electron transfer to molecular oxygen at or near the same site where antimycin A (AA) acts within the Q-cycle of Complex III (Fato et al., 2009; Gudz et al., 1997). C16:0 ceramide was also reported to inhibit the Complex IV activity in isolated liver mitochondria and induce ROS production (Zigdon et al., 2013). C16:0 ceramide also disrupts mitochondrial *β*-oxidation by inhibiting carnitine palmitoyltransferase 1 (CPT1), a key enzyme regulating the entry of long-chain fatty acids into mitochondria for oxidation. This inhibition results in a lipid overload state, where non-oxidized fatty acids are diverted into pathways generating more ceramides, perpetuating a self-amplifying cycle of metabolic stress and dysfunction (Serra et al., 2013). A recent report documented that pharmacologic accumulation of ceramide resulted in disruption in ETC and ROS production in human neurons (Kennedy et al., 2016). Crivelli et al. (2024) reported that elevated levels of 18:0, 22:0, and 24:1 ceramide reduced the ETC and increased ROS production in isolated synaptic and non-synaptic mitochondria from 5xFAD mice, a widely used Alzheimer’s disease model. This result resembles what it was found in recent reports documented that ceramide accumulation compromises mitochondria respiration in the cerebral cortex (Carr et al., 2023) (Figure 2).

### 3.3 Ceramides coordinate apoptosis and mitophagy

Ceramides have been implicated in the induction of apoptosis and mitophagy by several mechanisms. Ceramide accumulation can lead to mitochondrial and membrane permeabilization, triggering the release of cytochrome c into the cytoplasm favoring caspase-3-dependent apoptosis. Ceramides also activate apoptosis by depleting the anti-apoptotic proteins like Bcl-2 in neurons (Jazvinšćak Jembrek et al., 2015), inhibits the Akt/PKB survival pathway and activates the mitogen-activated protein kinases (MAPKs) (Iwayama and Ueda, 2013). As described, ceramides are capable of forming membrane channels in both planar phospholipid membranes and the outer membranes of isolated mitochondria, large enough to allow the passage of proteins released during mitochondrial outer membrane permeabilization (MOMP) (Fisher-Wellman et al., 2021). The anti-apoptotic protein Bcl-xL has been identified as a key regulator of this pathway, as it inhibits both MOMP *in vivo* and the formation of ceramide channels *in vitro* (Chang et al., 2015). During apoptosis, ceramides can affect mitochondria by ceramide-formed channels, favoring mitochondrial outer membrane permeability (MOMP), kinase activation, and inhibition of respiration (Stiban and Perera, 2015). Conversely, the ceramide-metabolite sphingosine-1-phosphate (S1P) inhibits apoptosis in endothelial cells (Bonnaud et al., 2007). Accordingly, a recent report documented that brain apoptosis faced an increase in the C16-ceramide levels potentially by the conversion from mitochondrial sphinganine and sphingomyelin (Mignard et al., 2020). Notably, C16-ceramide is enhanced in mitochondria when sphingomyelin levels are decreased in the MAMs microdomains (Mignard et al., 2020). These results propose that ceramide induces apoptosis by increasing mitochondrial Bax expression which supports the notion that ceramide assist channels formation. Also, ceramides seem to integrate a mutual dependent crosstalk between mitochondria – ER by MAMs microdomains during apoptosis regulating by C16-species (Figure 2).

Mitophagy and apoptosis are mutually regulated to secure cell integrity during neuronal death and survival. Maintenance of the mitochondrion pool is sensitive to both fusion and fission molecular machinery in a way to selectively identify and remove damaged mitochondria (Scheibye-Knudsen et al., 2015). Mitophagy, a form of autophagy, is responsible for the basal mitochondrial turnover that eliminates dysfunctional mitochondria. Notably, new evidence documented that mitochondria split into two separate types, one of which concentrates on energy production, the other on producing essential cellular building blocks (Ryu et al., 2024). Accordingly, mitochondrial fusion preserves the mitochondria for greater energy production during conditions of high metabolic activity, whereas fission facilitates the removal of damaged mitochondria via mitophagy (Scheibye-Knudsen et al., 2015). C18:0 ceramide specie is a stress-mediated mitophagy response which is mediated by the subcellular localization of CerS1 in damaged mitochondria vs. endoplasmic reticulum in the brain (Oleinik et al., 2023; Ryu et al., 2024). Recently, it was reported that mitophagy requires the subcellular interaction of CerS1 by the p17/PERMIT protein (17 kDa transporter) to secure proper mitochondrial quality control in the brain by mitophagy (Oleinik et al., 2023). Based on this model, CerS1 is transported to the outer mitochondrial membrane by the p17/PERMIT to recognize and induce C18:0 ceramide-mediated mitophagy in brain (Oleinik et al., 2023). In fact, CerS1 and its metabolic product, C18-ceramide (Dany and Ogretmen, 2015), induce non-apoptotic lethal mitophagy independent of Bax, Bal, or caspase activity in carcinoma cells (Sentelle et al., 2012). Interestingly, ectopic expression of CerS1 or treatment with C18-pyridinium-ceramide resulted in LC3-II formation and promoted its direct binding to ceramide on the mitochondrial membranes. This lipid-protein binding then allowed the mitochondria to be targeted by the LC3-II-containing autophagosome (Sentelle et al., 2012). Conversely, endogenous C16-ceramide generated by ceramide synthase 6 did not activate mitophagy-promoting function (Sentelle et al., 2012). These results support the role of ceramide signaling in mediating mitophagy through ceramide-LC3-II binding (Figure 2).

Mitophagy might be also regulated by acid sphingomyelinase (ASM), a ceramide metabolite enzyme. The ASM is activated by several cellular stresses implicated in both apoptosis and autophagy by modulating the balance between ceramide and its metabolic derivatives, sphingosine (Sph) and sphingosine-1-phosphate (S1P) (Lee et al., 2014). Precisely, ASM modulates the lysosomal nutrient-sensing complex (LYNUS), which integrate a mTOR and transcription factor EB (TFEB) mutual crosstalk. Justice et al. (2018) reported that ASM activity supports physiological mTOR signaling by preventing TFEB activation. Pharmacologic or genomic ASM inhibition enhanced nuclear translocation of TFEB and autophagic activity. Also, Oleinik et al. (2023) demonstrated that C18-ceramide promoted mitophagy through LC3B lipidation to form LC3B-II. In a recent paper, authors documented that oxidative stress targets ceramide to mitochondria by extracellular vesicles from the plasma membrane. Authors reported that elevation of ceramide by sphingomyelinase 2 and acid sphingomyelinase activity were target to mitochondria by ceramide-rich extracellular vesicles favoring cell death (Quadri et al., 2024). These results suggest that ceramides and ASM coordinate mitochondria and autophagosome membrane formation assisting mitophagy (Figure 2).

Defective mitophagy sensitive to ceramides has also been reported in PD (Vos et al., 2021). PINK (PTEN-induced putative kinase1)/Parkin pathway is the most well-characterized signaling that orchestrates mitochondrial degradation in PD (Doxaki and Palikaras, 2020). Under physiological conditions, the cytosolic PINK1 enters the mitochondria through the translocases of the outer and inner membranes complexes (Markaki et al., 2023). PINK1 is cleaved by mitochondrial processing peptidase and the presenilin-associated rhomboid-like protease and is finally completely degraded by an MG132-sensitive protease (Greene et al., 2012). Then, PINK1 is stabilized on the outer membrane and is activated by autophosphorylation, leading to the recruitment of cytosolic E3 ubiquitin ligase Parkin, which in turn, ubiquitinates several outer mitochondrial membrane proteins promoting mitochondrial (Sekine and Youle, 2018; Doxaki and Palikaras, 2020). In fact, homozygous loss of PINK1 or Parkin results in PD (Vos and Klein, 2022), supporting the role of PINK1 on mitochondria quality control. Of note, authors reported interaction between ceramides and the PINK1 protein affecting mitochondria function and mitophagy. Notably, defective PINK1 leads to decreasing beta-oxidation, increasing ceramide accumulation in mitochondria and mitophagy (Vos et al., 2021). Moreover, lower ceramide levels improved mitochondrial phenotypes in PINK1-mutant flies and PINK1-deficient patient-derived fibroblasts (Vos and Klein, 2022). Moreover, lower ceramide levels improved mitochondrial phenotypes in PINK1-mutant flies and PINK1-deficient patient-derived fibroblasts (Vos et al., 2021). Also, using other brain damage models, it has been described that intracerebral hemorrhage by hemin treatment increases the upregulation of ceramide synthase 6 (CerS6) and CerS6-derived C16 ceramide biosynthesis (Xu et al., 2024). Furthermore, CerS6 was found to have a detrimental effect on mitochondrial dynamics and function, while increasing apoptosis in neuronal cell culture. Additionally, CerS6 interacts with the sequestosome 1 protein, impairing mitophagy and contributing to mitochondrial dysfunction (Xu et al., 2024).

Finally, ceramide synthesis and accumulation in brain might be sensitive to metabolic triggers. A recent report documented that insulin significantly increased C16:1, C20, C24, and C24:1 synthesis in the cerebral cortex of ApoE4 mice model (Carr et al., 2023). Also, C18-ceramide production seems to modulate the argininosuccinate/fumarate/malate axis and mitophagy in neurodegenerative diseases. Authors reported that exogenous fumarate or malate inhibited mitophagy, leading to mitochondrial damage and sensorimotor abnormalities associated with aging in p17/PERMIT−/− mice (van Eijk et al., 2017). Conversely, C18-ceramide activates mitophagy and improved sensorimotor deficits in aged p17/PERMIT−/− mice (van Eijk et al., 2017).

These findings reinforce the link between ceramides species positively or negatively modulating mitophagy and mitochondrial dysfunction in neurodegenerative diseases.

## 4 Intermittent fasting (IF)

By definition, fasting is a voluntary deprivation of food and caloric beverages, for different purposes, ranging from religious (Galassi et al., 2018) or political reasons to obtaining health benefits. Alternating between periods of food restriction and periods of food intake is known as IF. IF has currently been proposed as a strategy for reducing and controlling body weight (Chair et al., 2022; Jospe et al., 2020; Templeman et al., 2021), as well as improving metabolic and cardiovascular health (Liu et al., 2022; Sutton et al., 2018).

There are various IF protocols, distinguished by the duration and frequency of fastin periods. In this review, we focus on the following protocols: time-restricted eating, alternate-day fasting and twice-per-week fasting (Koppold et al., 2024). During the time-restricted eating (TRE) (or time restricted feeding -TRF- in animal models) daily eating hours are reduced (Liu et al., 2022), allowing food intake ad libitum in windows ranging from 4 up to 10 h (14–20 h of fasting per day) (Lowe et al., 2020; Mayra et al., 2022; Wilkinson et al., 2020). Several authors do not properly consider TRE as a type of IF due to time of eating to certain hours each day, and it may not involve significant food deprivation compared to other types of fasting (Attinà et al., 2021; Fanti et al., 2021). Accordingly, alternate day fasting (ADF), also called every other day fasting (Alzoubi et al., 2021), involves 1 day of ad libitum feeding followed by a full day of fasting (Stekovic et al., 2019; Hoddy et al., 2014). In twice-per-week fasting (such as the so-called “5:2 diet”), fasting is performed on 2 non-consecutive days of the week, and the remaining 5 days are for habitual food intake (Scholtens et al., 2020). In both ADF and twice-per-week fasting regimen, caloric intake can be very low during fasting periods (less than 25% of daily caloric intake), which is referred to as modified (Hoddy et al., 2014; Koppold et al., 2024).

Despite variations in fasting protocols, alternating between fasting and regular eating leads to significant metabolic and hormonal adaptations of body physiology. In IF, the use of the body’s energy reserves, such as liver glycogen and adipose tissue triacylglycerols, is increased. After about 12 h of fasting, a metabolic switch occurs and the use of triacylglycerols increases (Mattson et al., 2018), leading to changes in brain metabolic activity.

Next, we will address the effect of IF on ceramides profiles assisting mitochondria function and dynamics.

## 5 IF regulates ceramides profiles and mitochondrial function and dynamics

### 5.1 Effect of IF on ceramides levels and lipid composition in the mitochondria membranes

**Figure 3:**
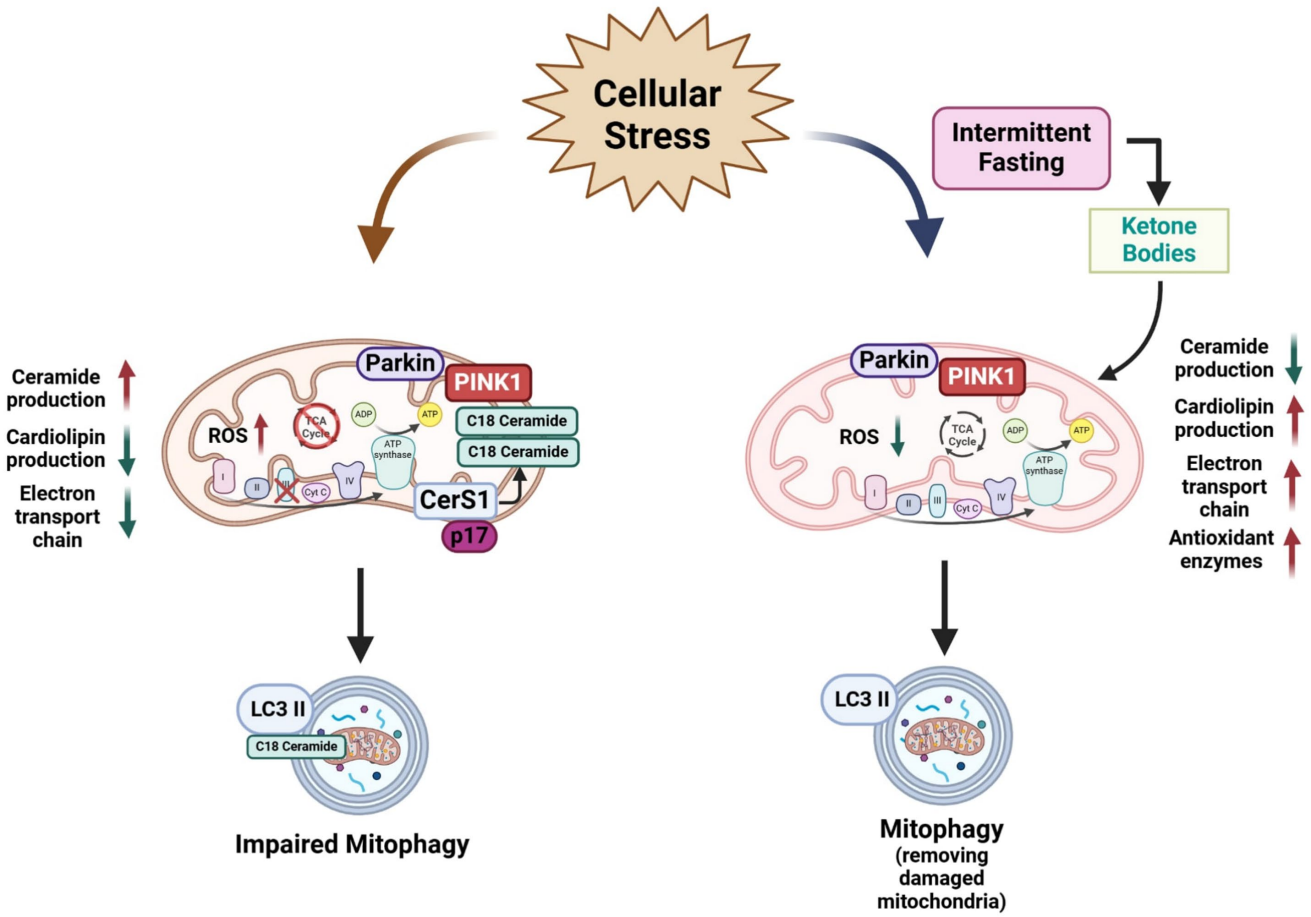
Schematic representation of the negative effects of ceramide in mitochondria physiology and mitophagy and a possible beneficial effect of intermittent fasting on mitochondria. Ceramide overproduction produces negative effects on mitochondria physiology such as an alteration in the mitochondrial respiratory chain function and ROS accumulation leading to oxidative stress. Furthermore, increasing levels of ceramide, especially C18:0 specie activates mitophagy. Conversely, IF improves mitochondrial function in several cell types by reducing ceramide production, reducing the ROS production by increasing antioxidant enzyme levels, production of cardiolipin levels in the mitochondria membrane, and improving the electron transport chain by enhancing mitochondrial oxidative metabolism and mitochondrial respiratory control rate. IF also can induce mitophagy to remove damaged mitochondria by several cellular insults. Scheme created in https://BioRender.com.

As mentioned above, IF and CR have been demonstrated to modify mitochondrial function, however, little is known regarding the effects of these dietary regimens on mitochondrial membrane composition. Cardiolipins are specific phospholipids of the mitochondrial inner membrane that participates in many aspects of its organization and function, hence promoting proper mitochondrial ATP production (Prola and Pilot-Storck, 2022). It is known that oxidative phosphorylation generates the electrochemical gradients to produce ATP synthesis, which occurs in the mitochondrial inner membrane and requires specific membrane morphology. Cardiolipins is an anionic molecule, with a dimeric molecular structure comprising three glycerol groups, two phosphate moieties, and four esterified fatty acyl chains, which are all bound to a compact polar head (Ikon and Ryan, 2017). Cardiolipin represents around 10–15% of mitochondrial membrane phospholipids and promotes the formation of mitochondrial cristae (Joubert and Puff, 2021), Cardiolipin also interacts with numerous other proteins involved in protein import, calcium transport, and cell death (Ghosh et al., 2020) (Figure 3).

A few reports have shown that CR or fasting could modulate cardiolipin biosynthesis or promote changes in mitochondria membranes. Amigo et al. (2017) demonstrated that isolated phospholipids from rat liver mitochondria from animals on ad libitum diet present an increased content of lipoperoxides and higher polarization. Conversely, CR diminished the lipoperoxides content and expression levels of the enzymes involved in cardiolipin biosynthesis and remodeling were found to be upregulated in CR-subjected animals. When mitochondrial membranes were fractionated, the outer membrane presented higher cardiolipin content, indicating a redistribution of this phospholipid mediated by phospholipid scramblase in CR animals. These changes were associated with the upregulation of DRP1-mediated mitochondrial fragmentation and autophagy proteins (Amigo et al., 2017) (Figure 3).

Menezes-Filho et al. (2019) reported that an overnight fasting (15 h) leads to a shift in the liver mitochondrial bioenergetics profile, with a reduction in ADP-stimulated and maximal respiration, lower membrane potential, and lower resistance to Ca2 + −induced mitochondrial permeability transition. Interestingly, 15 h fasting was able to increase cardiolipin quantities in isolated mitochondria from liver (Menezes-Filho et al., 2019). These findings show that dietary regimens could promote the remodeling of cardiolipin content in mitochondrial membranes.

### 5.2 Effect of IF on ceramides levels, ETC and ROS generation

Excessive glucose flux has been described as increasing the activity of the mitochondrial ETC, leading to ROS overproduction (Hallan and Sharma, 2016; Joaquim et al., 2022). IF protocols such as the ADF for one month was shown to reduce mitochondrial respiratory control in liver but not in the brain of young mice, showing a tissue-specific effect toward energy saving (Sorochynska et al., 2019). In normal aging, a decline in brain mitochondrial respiration and antioxidant enzymes has also been observed. Notably, aged mice exposed to ADF reduced oxidative stress and increased antioxidant enzyme levels, while having a modest effect in improving respiratory control (Bayliak et al., 2021). Furthermore, both ADF and time-restricted feeding reduced malondialdehyde accumulation in the mitochondria and increased complex I and III activity of the ETC in young and aged mice (Chen et al., 2024). Otherwise, IF is also beneficial to mitochondrial and peroxisomal lipid proteins homeostasis. For instance, IF restored ETC proteins and normalized the oxygen consumption rates of Complex I and II in a rat model of pulmonary arterial hypertension (PAH) (Almuraikhy et al., 2024). Mechanistically, it is documented that mitochondria electron transport chain genes are progressively sensitive to fasting. At 12 h of fasting, 14 genes were downregulated, whereas 6 genes, scattered over all 5 complexes were upregulated. At 24 h, 3 genes remained upregulated among 20 downregulated species. At 72 h of fasting, no fewer than 63% of the respiratory chain genes, including cytochrome C, were downregulated (Sokolović et al., 2007). This suggest that IF might modulate the mitochondrial electron transport chain at transcriptional level. (Figure 3).

IF has been demonstrated to confer survival benefits in several disease models (de Cabo and Mattson, 2019). Also, the role of IF has been studied in clinical trials. In a recent study in overweight and obese women with rheumatoid arthritis, IF decreased the oxidative stress marker as malondialdehyde levels in serum and, neutrophil-to-lymphocyte ratio together with an increase in catalase levels. Also, liver enzymes such as aspartate transaminase and alanine transaminase had a significant decrease in the IF group (Tavakoli et al., 2025). Such evidence suggests that IF might be integrated as an interventional approach to tackle oxidative stress and inflammatory profiles (Figure 3).

### 5.3 Effect of IF on apoptosis and mitophagy

IF positively modulates mitochondrial dynamics resulting in preserved mitochondrial health and cellular energetics (Diab et al., 2024). Authors reported that preservation of mitochondrial integrity could be attributed to suppressing excessive mitochondrial fission proteins by the upregulation of adipose triglyceride lipase Atg1, the rate limiting enzyme of triglyceride hydrolysis. The liberation of fatty acids by Atg1 during fasting contributes to enhanced mitochondrial oxidative capacity (Lettieri-Barbato et al., 2018). On the other hand, Real-Hohn et al. (2018) showed that IF increases mitochondrial mass compared with ad libitum animals. Furthermore, these authors found that IF group had a lower level of oxidative stress and greater oxidative capacity and promoting a balanced fusion-fission equilibrium and inducing mitophagy (Real-Hohn et al., 2018). McWilliams et al. (2018) also reported the effect of fasting on basal mitophagy in mouse tissues with high metabolic demand such as the brain, heart, eye, liver, kidney, and spleen. According, a high degree of mitophagy occurred in neuronal cells such as dopaminergic neurons and microglia (McWilliams et al., 2018). In high fat-fructose diet-induced liver and skeletal muscle injury model, Bastawy et al. (2024) showed that IF and metformin upregulated mitophagy-related genes such as PINK1, Parkin, and LAMP, attenuated the hepatic triglyceride content and decreased oxidative stress markers (Bastawy et al., 2024). During low energetic supply, it is expected that activation of the AMP-activated protein kinase (AMPK), which activates mitophagy. Conversely, mice lacking skeletal muscle AMPK subjected to fasting conditions showed that both mRNA and protein level of autophagy adaptor protein p62 was increased in fasted conditions. Moreover, Parkin protein level increased by more than two-fold in AMPK-MKO compared with wild-type mice (Bujak et al., 2015) (Figure 3).

Finally, calorie restriction (CR) has also probed to modulates mitophagy. Long-term CR reduces mitochondrial biogenesis and mitophagy in liver, as measured by quantitative proteomics (Price et al., 2015). Also, CR was able to become susceptibility to mitophagy, together with an increase of both Pink1 mRNA and an increase of Drp1 protein (Zhao et al., 2013).

This evidence suggests that intermittent fasting, calorie restriction or fasting induces mitophagy and mitophagy-related markers, whereby it could be concluded that restrictive interventions have a role in the regulation of health by inducing mitochondria autophagy in different organs of the body (Mehrabani et al., 2020).

## 6 Conclusion

The current review provided experimental evidence supporting that dysregulation of ceramides metabolism assists neurodegenerative disorders through mitochondria dysfunction. Here, we propose that ceramides susceptibilizes to neurodegeneration by affecting mitochondria membrane composition, respiratory chain, ROS dynamics, ATP production and mitophagy-apoptosis in brain. We conceive that dysregulation of ceramide metabolism is sensitive to ultra-processed foods intake normally exposed during the current day life. Elucidating how ceramides exert their effects can enhance pathophysiological understanding of neurodegenerative disorders and constitute potential biological targets for novel drugs and diagnostic and prognostic biomarkers.

A major potential conclusion of our review is the effect of IF on neurodegeneration. IF has been shown to be an effective strategy to control body weight and improve several metabolic parameters. However, its application in the context of neurodegenerative disorders may be limited by some contrasting or inconclusive results, representing a constantly evolving area of research. We provide evidence documented that IF positively targets mitochondria at three levels: membrane lipid composition, respiratory chain and ROS generation and mitophagy-apoptosis. In this context, IF might be integrated as a future non-pharmacological strategy supporting integral treatment of neurodegenerative disorders. Additionally, a potential combination of IF with pharmacological treatments may enhance therapeutic intervention and efficacy by targeting metabolic and inflammatory pathways which normally accounts during physiological brain aging.

By now, research on IF and its role on neurodegeneration faces significant challenges, including a limited number of studies addressing IF protocols on neurodegeneration. This heterogeneity complicates the ability to draw definitive conclusions or recommend a standardized protocol mainly in preclinical models. Future research should prioritize the development of consistent IF protocols in animal models and characterize selective molecular targets. Addressing these challenges will identify selective cellular and molecular pathways linking ceramides metabolism to neurodegenerative disorders, which are essential for translating IF into sustainable and broadly applicable strategies in clinic for improving brain health potentially focusing on neurodegeneration.
